# Feasibility of mHealth to Support Self-Management in Older Adults Living with HIV

**DOI:** 10.1093/geroni/igaf122.2678

**Published:** 2025-12-31

**Authors:** Gwang Suk Kim, Layoung Kim, Sooyoung Kwon, Seoyoung Baek

**Affiliations:** Yonsei University College of Nursing, Seoul, Republic of Korea; The University of Suwon, Hwaseong, Republic of Korea; Graduate School of Yonsei University, Seoul, Republic of Korea; Korea Armed Forces Nursing Academy, Daejeon, Republic of Korea

## Abstract

HIV infection is a chronic disease requiring lifelong management. Social stigma and healthcare discrimination limit access to health resources, making mobile health (mHealth) a promising intervention. However, concerns exist regarding the applicability of mHealth among older adults given potential barriers and engagement levels. This study assessed the feasibility of mHealth for older people living with HIV (PLWH). A one-arm, prospective, fully non-face-to-face mHealth trial was conducted with 24 PLWH aged ≥60 years. Participants used an mHealth app, “ESSC (Excellent Self-Supervised HIV Care),” which provided health-related information; allowed self-recording of medication adherence, mental diary, and sexual life; and enabled Q&A and comments interactions. After baseline assessments of demographics and HIV-related health, participants used the app for four weeks. Cumulative usage data were analyzed. All 24 participants were successful installing the app independently. Participant’s median age was 63.88 years (range: 60–82), and all were male. Based on usage, 7 participants (29.2%) were non-users (≤2 days), while 17 (70.8%) were users (≥24 days). Among users, the 28-day average included 33.6 daily medication logs, 14.2 mental diary entries, 7.6 sexual life records, 1.5 Q&A interactions, and 9.8 comments. Notably, users exhibited significantly higher baseline depression scores (6 vs. 0), higher stigma levels (8.7 vs. 7.0), and lower health-related quality of life (70 vs. 90) than non-users. This study demonstrated the feasibility of mHealth interventions for older PLWH. Findings suggest that mHealth adoption may be particularly beneficial for older PLWH with poorer emotional health, highlighting the need for targeted interventions to enhance usability.

